# Genetic Selection of Low Fertile *Onchocerca volvulus* by Ivermectin Treatment

**DOI:** 10.1371/journal.pntd.0000072

**Published:** 2007-08-30

**Authors:** Catherine Bourguinat, Sébastien D. S. Pion, Joseph Kamgno, Jacques Gardon, Brian O. L. Duke, Michel Boussinesq, Roger K. Prichard

**Affiliations:** 1 Institute of Parasitology, McGill University, Sainte Anne-de-Bellevue, Quebec, Canada; 2 Laboratoire de Neuroparasitologie et Neuroépidémiologie Tropicale, Faculté de Médecine, Limoges Cedex, France; 3 National Onchocerciasis Task Force Cameroon, Yaoundé, Cameroon; 4 UR 24 Epidémiologie et Prévention, Institut de Recherche pour le Développement, La Paz, Bolivia; 5 River Blindness Foundation, Lancaster, United Kingdom; 6 UR 24 Epidémiologie et Prévention, Département Sociétés et Santé, Institut de Recherche pour le Développement, Paris Cedex, France; New York Blood Center, United States of America

## Abstract

**Background:**

*Onchocerca volvulus* is the causative agent of onchocerciasis, or “river blindness”. Ivermectin has been used for mass treatment of onchocerciasis for up to 18 years, and recently there have been reports of poor parasitological responses to the drug. Should ivermectin resistance be developing, it would have a genetic basis. We monitored genetic changes in parasites obtained from the same patients before use of ivermectin and following different levels of ivermectin exposure.

**Methods and Findings:**

*O. volvulus* adult worms were obtained from 73 patients before exposure to ivermectin and in the same patients following three years of annual or three-monthly treatment at 150 µg/kg or 800 µg/kg. Genotype frequencies were determined in β-*tubulin*, a gene previously found to be linked to ivermectin selection and resistance in parasitic nematodes. Such frequencies were also determined in two other genes, *heat shock protein 60* and *acidic ribosomal protein*, not known to be linked to ivermectin effects. In addition, we investigated the relationship between β-*tubulin* genotype and female parasite fertility. We found a significant selection for β-*tubulin* heterozygotes in female worms. There was no significant selection for the two other genes. Quarterly ivermectin treatment over three years reduced the frequency of the β-*tubulin* “aa” homozygotes from 68.6% to 25.6%, while the “ab” heterozygotes increased from 20.9% to 69.2% in the female parasites. The female worms that were homozygous at the β-*tubulin* locus were more fertile than the heterozygous female worms before treatment (67% versus 37%; *p* = 0.003) and twelve months after the last dose of ivermectin in the groups treated annually (60% versus 17%; *p*<0.001). Differences in fertility between heterozygous and homozygous worms were less apparent three months after the last treatment in the groups treated three-monthly.

**Conclusions:**

The results indicate that ivermectin is causing genetic selection on *O. volvulus*. This genetic selection is associated with a lower reproductive rate in the female parasites. We hypothesize that this genetic selection indicates that a population of *O. volvulus*, which is more tolerant to ivermectin, is being selected. This selection could have implications for the development of ivermectin resistance in *O. volvulus* and for the ongoing onchocerciasis control programmes.

## Author Summary

*Onchocerca volvulus* is the causative agent of onchocerciasis, or “river blindness”. Ivermectin has been used for mass treatment of onchocerciasis for up to 18 years, and recently there have been reports of poor parasitological responses to the drug and evidence of drug resistance. Drug resistance has a genetic basis. In this study, genetic changes in β-*tubulin*, a gene associated with ivermectin resistance in nematodes, were seen in parasites obtained from the patients exposed to repeated ivermectin treatment compared with parasites obtained from the same patients before any exposure to ivermectin. Furthermore, the extent of the genetic changes was dependent on the level of ivermectin treatment exposure. This genetic selection was associated with a lower reproductive rate in the female parasites. The data indicates that this genetic selection is for a population of *O. volvulus* that is more tolerant to ivermectin. This selection could have implications for the development of ivermectin resistance in *O. volvulus* and for the ongoing onchocerciasis control programmes. Monitoring for the possible development and spread of ivermectin resistance, as part of the control programmes, should be implemented so that any foci of resistant parasites can be treated by alternative control measures.

## Introduction

*Onchocerca volvulus* is the filarial nematode, transmitted by *Simulium* flies, that causes human onchocerciasis, or “river blindness”. It is estimated that 37 million people, mostly in Africa, are infected with this worm [1]. At present, ivermectin (IVM, Mectizan) is the only safe drug available for mass treatment of onchocerciasis. IVM, administered at the standard dose of 150 µg/kg, has a rapid effect on the embryonic stage of the parasite, the microfilariae (mf), which cause most of the ocular and cutaneous manifestations of the disease. As a result of this microfilaricidal effect, the skin microfilarial loads decrease by 95–99% within one month after treatment. The drug also blocks the production of new mf by the adult female worms, who only resume mf release 3–6 months after treatment. This “embryostatic effect” of IVM explains why the mf loads remain at very low levels for up to one year. Furthermore, IVM treatments repeated at 1- to 3-monthly intervals have some, though moderate, effect on the longevity of the adult worms (“macrofilaricidal effect”) [2,3].

The drug, when given repeatedly, is therefore acting on at least three components of parasite fitness: reproduction, microfilarial survival and adult parasite lifespan, which together affect morbidity and the intensity of transmission. Due to the limited macrofilaricidal effect of the drug, treatments must be repeated and sustained. Endemic communities in Africa receive annual IVM treatment, while those of Latin America receive semi-annual treatments. To date, more than 400 million treatments have been distributed in Africa [4], with some individuals having received up to 18 annual treatments.

Due to this enormous drug pressure on the parasite, there is a risk of resistance of *O. volvulus* to the drug [5–7]. This concern is justified by reports of suboptimal responses to IVM from Sudan [8] and Ghana [9,10], although in the former report reduced immune responsiveness in some of the treated people has been suggested as a possible explanation for the suboptimal responses to IVM. And in the study in Ghana the poor responses have been attributed to the parasites, with adult female worms resuming microfilarial production earlier after treatment than classically described. More recently, another report in Ghana [11] shows the first unequivocal parasitological and epidemiological evidence of ivermectin resistance in *O. volvulus* populations.

In addition to this evidence of IVM resistance, changes in the genetic structure of *O. volvulus* populations, associated with IVM treatments, have been observed in parasites from Ghana [12–16]. These changes occurred particularly on the β-*tubulin* gene [16,17], which has been associated with IVM resistance in the sheep intestinal nematode *Haemonchus contortus* [17]. However, in these previous studies, *O. volvulus* from IVM-naïve and -treated human populations were collected from different individuals in different communities.

It is important to assess whether the genetic changes reported in *O. volvulus* are associated with a reduced response to IVM in any of the three effects of IVM on parasite fitness, described above. Furthermore, to eliminate the possibility that differences in genotype frequencies between IVM-naïve and -treated populations could be due to geographical effects, due to separate individuals and communities being sampled, it is important to assess whether changes in genetic frequency could occur in parasites collected from the same individuals before and after exposure to IVM. Genetic changes clearly associated with treatment, which could not possibly be associated with other covariates, would provide unequivocal evidence of genetic selection by IVM on *O. volvulus*. Such treatment-induced selection would be heritable. Heritable genetic changes that could reduce the susceptibility of *O. volvulus* to any of the effects of IVM on the parasite could have long-term consequences for the control of onchocerciasis because there is currently no alternative drug available for mass treatment of this disease.

In a previous study [18], we reported that in an IVM-naïve *O. volvulus* population from Cameroon, adult female worms presenting a homozygous genotype for β-tubulin were more fertile than adult worms that were heterozygous at this locus. In the present study, we have analyzed genetic characteristics (β-*tubulin* gene and two control genes, *heat shock protein 60* (hsp60) and *acidic ribosomal protein* (ARP)) and phenotypic characteristics (female worm fertility) of parasites collected, in the same individuals, before and after 4 or 13 IVM treatments over a three-year period. These treatments were administered as part of a clinical trial conducted in Central Cameroon. The main objective of this trial was to assess the effects of different regimens of IVM treatment on the mortality of *O. volvulus* adult worms, and the results of this phase have been published elsewhere [3]. In the second phase, results of which are presented in this paper, we evaluated whether repeated treatment with IVM led to (a) genetic changes in the adult worm population and (b) any modification of the relationship between β-*tubulin* genotype of the female worms and their reproductive status.

## Methods

### Study Area, Study Design and Selection of Patients

The study was carried out in the Mbam Valley, a region hyper-endemic for onchocerciasis, located in the Central province of Cameroon, where no IVM had been distributed at the beginning of the study and where no vector control activities have ever been performed. In this area, before the introduction of IVM, the intensity of infection in the population, as expressed by the Community Microfilarial Loads (CMFL) [19] ranged between 10 and 114 mf per skin snip (mf/ss) [3]. The full details of the clinical trial, which was approved by the Cameroonian Ministry of Public Health and by Merck and Co., the manufacturer of IVM (Mectizan), have been published elsewhere [3]. The study also subsequently received approval from the institutional review board of McGill University. Briefly, 657 individuals were selected using the following inclusion criteria: men between 18 and 60 years old, with at least two palpable nodules during the preliminary examination but otherwise in good health, who had not received any filaricidal treatment within the five previous years, and who agreed to participate in the trial by signing an informed consent form. These patients were randomly allocated to one of the four following IVM treatment groups: 150 µg/kg body weight/year (standard group; group 1); 150 µg/kg/three-monthly (group 2); 800 µg/kg/year (group 3); and 800 µg/kg/three-monthly (group 4). Over the three-year study period, patients received either 4 or 13 IVM treatments.

### Collection of Nodules and Parasitological Examination

In order to assess the macrofilaricidal effect of IVM on *O. volvulus*, adult worms were collected, by nodulectomy, at the outset of the trial (before the first IVM dose was administered) and once again after three years of treatment in the four different treatment groups described above. The protocol used for parasite collection was identical for the two rounds of nodulectomy. Just before each round of nodulectomy, each person was carefully examined and all the sites on their body where a nodule or a group of nodules was palpable were recorded on a body chart. Subsequently, one of the sites was selected at random and all the nodules located at this site were removed from each person. The site selected for the second nodulectomy was one of those recorded at the outset of the study so that the worms collected at that time had probably been subjected to the IVM treatments administered over the previous three years. Just after the nodulectomy, all the nodules collected were immersed in fixative (70% ethanol, 20% water, 10% glycerol). One of the nodules was used for histological examination, as previously described [3], to evaluate the status of the worms. Any additional nodules (“extra nodule”) from the excision site were stored in the fixative at room temperature and available for genotyping and phenotyping.

### Selection of Nodules for Genotyping and Phenotyping

Of the 657 individuals selected before treatment, 290 had more than one nodule at the first nodulectomy site, and thus at least one “extra nodule” available after the histological examination. Similarly, of the 541 patients present at the second round of nodulectomy (following three years of treatment), 156 had at least one extra nodule available. Patients included in the present study were selected taking into account our objectives, which were to assess the genotypes of three polymorphic genes, including β-*tubulin*, in the adult worms, and any relationship between the genotype of female parasites and their reproductive status, before and after IVM treatment. To make the comparison more sensitive, we performed the genotyping and the phenotyping only on parasites obtained from those people for whom “extra nodules”, containing at least one adult worm, had been collected at both nodulectomy rounds (pre-treatment and after three years of repeated treatments). The total numbers of individuals who met these inclusion criteria were 18 in group 1, 16 in group 2, 22 in group 3 and 17 in group 4. Thus, the analyses were performed on the nodules collected from 73 individuals.

### Procedure for Phenotyping the Female Reproductive Status

This procedure has been described previously [18]. In 2002, the nodules were washed with phosphate buffered saline (PBS) for 24 h with regular changes of medium in order to remove all residues of fixative. The nodules were then digested in collagenase [20]. Worms were collected and stored individually in labelled Eppendorf tubes, which were frozen at −80°C. Each female worm was phenotyped by microscopical examination of its reproductive status in terms of the presence of mf and embryos. Three phenotypes were defined: (a) non-fertile females, i.e. worms with empty reproductive organs, (b) females with low fertility, in which the reproductive organs contained only a few embryos, but no mf, and (c) fully fertile females, in which the reproductive organs were full of mf and embryos.

### Procedure for Genotyping

After the phenotyping, each worm was disrupted and its DNA was extracted using a Dneasy kit (Qiagen Inc., Mississauga, Canada). *Heat shock protein 60* (*hsp60*) (GenBank, AF121264), which is a molecular chaperone that participates in the folding of proteins, was chosen as a control gene. It was known to be polymorphic and previously found not to be selected by IVM treatment in *O. volvulus* [16]. Two polymorphs (“A” and “G”) were found in the *hsp60* gene partial sequence analyzed. The region analyzed started at position 214 on the cDNA and included 100 bp in the exon, followed by 276 bp in the intron. The A/G polymorphism was located in the intron region. The fragment of 376 bp was amplified by PCR from individual adult worms with the primers 5′CAA TCA TGG GGA AGT CCA AAG 3′ and 5′CTC AAA ACC TTC CTT TGC AAT 3′ at Tm = 53°C. PCR products were sequenced with the *hsp60* anti-sense primer using the 3730XL DNA Analyzer system (McGill University/Genome Quebec Innovation Centre). Platinum Taq DNA polymerase High Fidelity (Invitrogen) was used in the PCR reaction to avoid introduction of error during amplification. Each individual chromatogram was analyzed with Sequencher 4.7 software (Gene Codes Corporation, Ann Arbor, MI, USA), to detect the homozygotes AA and GG and the heterozygotes AG.

The *acidic ribosomal protein* (*ARP*) gene (GenBank, AI130565), which is involved in protein synthesis, was chosen as a second control because it was expected to be polymorphic [21] and not known to be sensitive to IVM treatment. Two polymorphs (“C” and “T”) were found in the acidic ribosomal protein gene partial sequence analyzed. The region of interest was from 1270 bp to 1488 bp of the complete gene. It was amplified by PCR from individual adult worms with the primers 5′ TGA AAA ACT GCT ACC GCA TA 3′ and 5′ AAA TTT TCG TTG GAA TTT GC 3′ at Tm = 54°C. PCR products were analyzed by restriction fragment length polymorphism, based on C/T polymorphism apparent in the EST database, using the restriction enzyme Mnl 1 for 2 hours, and subjected to electrophoresis on a 12% polyacrylamide gel (39∶1) for 2 hours at 130 V, stained with ethidium bromide and visualized using an ABI Imager (Bio-Rad, Hercules, CA, USA).

Two alleles (“a” and “b”) have been described for β-*tubulin* [16]. These two alleles have three single nucleotide polymorphisms in an exon region. These differences lead to changes in three amino acids in the putative protein sequence. The worms were genotyped individually for β-*tubulin* (GenBank, F019886) by PCR amplification followed by amplicon length analysis [17].

### Statistical Analysis

The aim of the analysis was to assess whether a variety of covariates related to the worm, nodule or patient characteristics were associated with three different dependent variables: (a) the inability to genotype some of the worms from the preserved nodules; (b) the frequency of the various polymorphs analyzed; and (c) the degree of fertility of the worm. We considered the five following covariates: the age of the patient at the outset of the trial (continuous variable); the CMFL in the village of residence of the patient, defined in four categories: 10–40, 41–60, 61–70, and 71–114 mf/ss; the treatment group (for analysis of the worms collected post-treatment: 150 µg/kg/year, 150 µg/kg/three-monthly, 800 µg/kg/year, and 800 µg/kg/three-monthly); the total number of females in the nodule; and the total number of palpable nodules on the patient at the outset of the trial. In addition, we also assessed the degree of fertility in relation to the genotype of the worms and to the total number of males in the nodule.

### Study of Possible Bias in Female Worms That Could Not Be Genotyped

The procedure for genotyping the worms failed with a significant number of worms obtained from the nodules that had been preserved at room temperature for 5 to 8 years. To test whether this inability to genotype some worms could be explained by sampling biases, we assessed, using multivariate logistic regression, whether the success in genotyping the worm (genotyped vs. non-genotyped status) was associated with one or the other of the possible covariates quoted above. All regressions analyses were performed using Stata v9.0 (Stata Corporation, TX, USA), where parameters were estimated using the cluster option [22] accounting for intra-nodular correlation.

### Changes in Genotypic Frequencies

Hardy-Weinberg equilibrium was tested using the χ^2^ test, unless the sample size was small. In this case, Fisher's exact test was used. The genotypic frequencies before and after treatment were compared using Fisher's exact test. To evaluate whether some host covariates or village characteristics may have influenced the heterozygosity of the worms, the association between heterozygous status and the five main possible covariates quoted above was assessed separately on pre- and post-treatment data, by multivariate logistic regressions. Potential intra-nodule clustering was accounted in the regression models.

### Relationship between Genotype and Fertility of Female Worms Before and After Treatment

Logistic regression models were used to analyze the independent variables associated with the fertility of the female worms before and after treatment. The dependent variable “fertility” was defined, for this analysis, using two categories: no or low fertility versus high fertility [18]. This choice is based on the fact that only worms with mf have the possibility of having their progeny transmitted, at the time of sampling, and this may be relevant to the possible transmission of any “resistant” genotypes. However, any treatment group effect on fertility status could be due to either treatment frequency or to the fact that the worms were collected three months after the last treatment in the three-monthly treated groups (groups 2 and 4) and twelve months post-treatment in the annual groups (groups 1 and 3). The possible covariates in the model included the five host-related independent variables defined above, and two other independent variables: the genotype of the worm at the β-*tubulin* locus (homozygous versus heterozygous), and the total number of males present in the nodule. Here again, the intra-nodule clustering was considered in the logistic regressions. The χ^2^ and Fisher's exact test analyses were performed using VassarStats (http://faculty.vassar.edu/lowry/VassarStats.html).

## Results

A total of 73 patients provided one nodule at the outset of the trial and one nodule after treatment. A total of 367 worms (248 females, 119 males) were isolated from the 73 nodules collected before treatment, and 224 worms (153 females, 71 males) were extracted from the 73 nodules provided by the same hosts after three years of repeated treatment. Details on the numbers of worms analyzed in the different treatment groups are given in Tables 1 and 2.

**Table 1:** Number of Nodules and Worms Collected and Genotyped Before Ivermectin Treatment in 73 Patients Who Provided Pre- and Post-Treatment Nodules.

Rx group	No. Nod.	Female worms					Male worms			
		No.worms (No./Nod.)	β-tubulin (%)	hsp60 (%)	ARP (%)	No. Phen.	No. worms (No./Nod.)	β-tubulin (%)	hsp60 (%)	ARP (%)
150*1	18	67 (3.72)	52 (77.6)	46 (68.7)	57 (85.1)	67	34 (1.89)	12 (35.3)	16 (47.1)	33 (97.1)
150*4	16	49 (3.06)	35 (71.4)	33 (67.3)	37 (75.5)	49	28 (1.75)	12 (42.9)	11 (39.3)	26 (92.9)
800*1	22	67 (3.05)	45 (67.2)	53 (79.1)	56 (83.6)	66	28 (1.27)	17 (60.7)	14 (50.0)	28 (100.0)
800*4	17	65 (3.82)	51 (78.5)	55 (84.6)	59 (90.8)	65	29 (1.71)	15 (51.7)	15 (51.7)	27 (93.1)
Total	73	248 (3.40)	183(73.8)	187 (75.4)	209 (84.2)	247	119 (1.63)	56 (47.1)	56 (47.1)	114 (95.8)

Rx group = treatment group (dose μg/kg * frequency per year); No. Nod. = number of nodules; No. worms = number of worms; No./Nod. = number of worms per nodule; β-tubulin = number of worms genotyped for β-tubulin; hsp60 = number of worms genotyped for hsp60; ARP = number of worms genotyped for acidic ribosomal protein; No. Phen. = number of worms phenotyped for fecundity.

**Table 2:** Number of Nodules and Worms Collected and Genotyped After Ivermectin Treatment in 73 Patients Who Provided Pre- and Post-Treatment Nodules.

Rx group	No. Nod	Female worms					Male worms			
		No. worms (No./Nod.)	β-tubulin (%)	hsp60 (%)	ARP (%)	No. Phen.	No. worms (No./Nod.)	β-tubulin (%)	hsp60 (%)	ARP (%)
150*1	18	41 (2.28)	32 (78.0)	24 (58.5)	31 (75.6)	39	22 (1.22)	5 (22.7)	10 (45.5)	22 (100.0)
150*4	16	35 (2.19)	21 (60.0)	17 (48.6)	28 (80.0)	34	15 (0.94)	4 (26.7)	5 (33.3)	12 (80.0)
800*1	22	47 (2.14)	27 (57.4)	28 (59.6)	39 (83.0)	45	23 (1.05)	13 (56.5)	10 (43.5)	22 (95.7)
800*4	17	30 (1.76)	18 (60.0)	20 (66.7)	26 (86.7)	29	11 (0.65)	4 (36.4)	4 (36.4)	11 (100.0)
Total	73	153 (2.10)	98 (64.1)	89 (58.2)	124 (81.0)	147	71 (0.97)	26 (36.6)	29 (40.8)	67 (94.4)

Rx group = treatment group (dose μg/kg * frequency per year); No. Nod. = number of nodules; No. worms = number of worms; No./Nod. = number of worms per nodule; β-tubulin = number of worms genotyped for β-tubulin; hsp60 = number of worms genotyped for hsp60; ARP = number of worms genotyped for acidic ribosomal protein; No. Phen. = number of worms phenotyped for fecundity.

### Study of the Independent Variables That Could Be Associated with the Inability to Genotype the Worms for the β-*tubulin* Gene

We previously showed, in a sample of 320 female worms collected before treatment as part of the same trial, that the 90 worms that could not be genotyped for β-*tubulin* did not differ significantly, with regard to several host independent variables, from the 230 worms that could be genotyped [18]. Similar results were obtained when comparing the 65 non-genotyped females to the 183 genotyped ones, and the 63 non-genotyped males to the 56 genotyped males, collected before treatment, from the 73 people from whom nodules could be analyzed both before and after treatment.

The proportion of female worms that could not be genotyped for β-*tubulin* was significantly higher after treatment (respectively 26.2% and 35.9% before and after treatment; *p* = 0.043). Among the 153 female worms collected after treatment, 55 could not be genotyped. According to multivariate logistic regression, the “genotyped” status was not associated with any of the five covariates included in the analysis. The proportion of non-genotyped male worms did not differ significantly before and after treatment (respectively 52.9% and 63.4%; *p* = 0.17). After treatment, we observed that a significantly higher proportion of male worms could be genotyped in the 800 µg/kg/year treatment group (OR = 3.97 (95% CI, 1.05–15.08); *p* = 0.043) compared to the standard group. None of the other independent variables differed significantly between the genotyped and the non-genotyped male worms. Taken together, these results do not provide evidence of bias between the 45 non-genotyped and 26 genotyped male worms according to the tested covariates.

### Genotypic Frequencies for the *Heat Shock Protein 60* Gene

#### Male worms

Amongst the 56 pre-treatment male worms that could be genotyped for this gene, the proportions of worms showing AA, AG and GG genotypes were 8.9%, 32.1% and 59.0%, respectively. This gene was in Hardy-Weinberg equilibrium before treatment. After treatment, among the 29 genotyped males, the corresponding proportions were 6.9%, 34.5% and 58.6%. Fisher's exact test did not demonstrate any significant difference in genotypic frequencies between pre- and post-treatment male worms (*p* = 0.99). It was not possible to determine whether treatment frequency affected male worm hsp60 genotype after treatment because the sample size was too small.

#### Female worms

Amongst the 187 pre-treatment female worms that could be genotyped, the proportions of AA, AG and GG were, respectively, 9.1%, 35.3% and 55.6%. This gene was in Hardy-Weinberg equilibrium before treatment. After treatment, among the 89 genotyped females, the corresponding proportions were 6.8%, 43.8% and 49.4%. Fisher's exact test did not demonstrate any significant difference in genotypic frequencies between pre- and post-treatment female worms (*p* = 0.37). It was possible to pool the female worms based on frequency of treatment because there were no significant differences between groups 1 and 3 before treatment (*p* = 0.96) and after treatment (*p* = 0.12), and between the groups 2 and 4 before treatment (*p* = 0.055) and after treatment (*p* = 0.40). There were no significant differences in the hsp60 genotype frequencies between the pooled annual treatment groups before and after treatment (*p* = 0.11) and between the three-monthly treatment groups before and after treatment (*p* = 0.23).

### Genotypic Frequencies for the Acidic Ribosomal Protein Gene

#### Male worms

Amongst the 114 pre-treatment male worms that could be genotyped for this gene, the proportions of worms showing CC, CT and TT genotypes were 4.4%, 27.2% and 68.4%, respectively. After treatment, among the 67 genotyped males, the corresponding proportions were 0%, 28.4% and 71.6%. Fisher's exact test did not demonstrate any significant difference in genotypic frequencies between pre- and post-treatment male worms (*p* = 0.27). Hardy-Weinberg analysis was not conducted on the acidic ribosomal protein gene because it has multiple copies in eukaryotes with copies occurring at different loci [21]. It was possible to pool the male worms, in frequency of treatment groups, because there were no significant differences between groups 1 and 3 before treatment (*p* = 0.47) and after treatment (*p* = 0.09), and between groups 2 and 4 before treatment (*p* = 1) and after treatment (*p* = 0.59). There were no significant differences in ARP genotypes frequencies between the pooled annual treatment groups before and after treatment (*p* = 0.51) and between the three-monthly treatment groups before and after treatment (*p* = 0.19).

#### Female worms

Amongst the 209 pre-treatment female worms that could be genotyped, the proportions of CC, CT and TT were, respectively, 0%, 89.0% and 11.0%. After treatment, among the 124 genotyped females, the corresponding proportions were 0.8%, 90.3% and 8.9%. Fisher's exact test did not demonstrate any change in genotype frequencies between pre- and post-treatment female worms (*p* = 0.38). It was possible to pool the female worms in frequency of treatment groups because there were no significant differences between groups 1 and 3 before treatment (*p* = 0.39) and after treatment (*p* = 0.08), and between groups 2 and 4 before treatment (*p* = 0.50) and after treatment (*p* = 0.61). There were no significant differences in ARP genotype frequencies between the pooled annual treatment groups before and after treatment (*p* = 0.59) and between the three-monthly treatment groups before and after treatment (*p* = 0.77).

### Genotypic Frequencies for the β-*tubulin* Gene

#### Male worms

Amongst the 56 pre-treatment male worms that could be genotyped, the proportions of homozygous allele a (aa), heterozygotes (ab) and homozygous allele b (bb) were, respectively, 92.9%, 5.4% and 1.8%. After treatment, among the 26 genotyped males, the corresponding proportions were 96.2%, 3.8% and 0%. No change in genotypic frequencies between pre- and post-treatment male worms was found, using Fisher's exact test. Before treatment, the 56 males were in Hardy-Weinberg equilibrium (Fisher's exact test, *p* = 0.99).

#### Female worms

**Figure 1:**
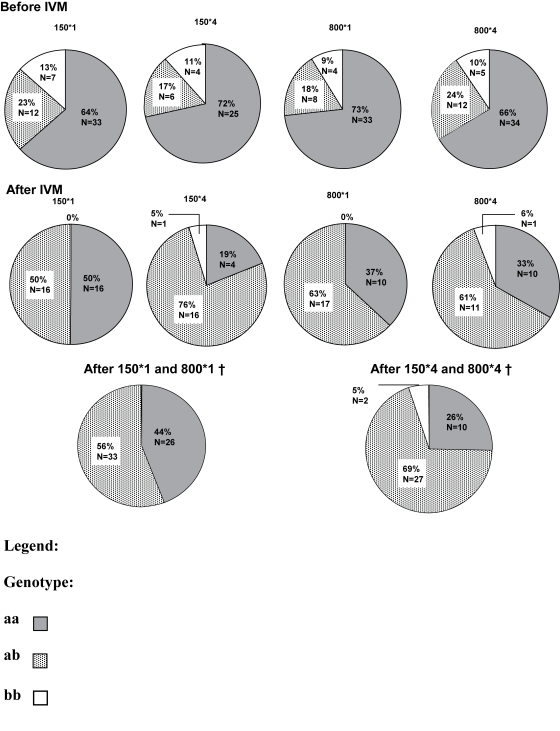
β-*tubulin* Genotype of Female Worms. β-tubulin genotype of female worms, that could be genotyped, before and after different doses of ivermectin. IVM = ivermectin treatment. † Pooled by treatment frequency.

Before treatment, there was no difference in genotype frequency (χ^2^ = 0.036; *p* = 0.98) between any of the groups. The 183 females analyzed before treatment were not in Hardy-Weinberg equilibrium (χ^2^ = 26.5; *p*<0.0001), with an excess of homozygotes. Amongst the female worms, the treatment led to an increase in the proportion of β-tubulin heterozygous worms (Figure 1). The reduction in the proportion of homozygous aa worms for β-tubulin was most marked in the 150 µg/kg three-monthly group (reduction rate in the proportion of aa worms: 73.6%). We also observed the same pattern of reduction in aa worms, though less dramatic, in the other treated groups (21.9%, 49.3% and 50.0%, respectively, in groups 1, 3 and 4). Conversely, the frequency of heterozygotes increased dramatically among the female worms collected in the group treated at 150 µg/kg three-monthly and also markedly in the other groups (χ^2^ test, *p*<10^−11^). It was possible to pool the female worms in frequency of treatment groups because there were no significant differences between groups 1 and 3 before treatment (*p* = 0.60) and after treatment (*p* = 0.43), and between groups 2 and 4 before treatment (*p* = 0.78) and after treatment (*p* = 0.72). After treatment, dose rate (150 or 800 µg/kg) per se did not have an effect on β-*tubulin* genotype frequency. However, there was a significant difference (Fisher's exact test, *p* = 0.047) between the pooled annual treatment groups with respectively 44.1%, 55.9% and 0% of aa, ab and bb and the three-monthly treatment groups with respectively 25.6%, 69.2% and 5.1% of aa, ab and bb. Hence treatment frequency, or the total number of treatments given within the three years, did have a significant effect on the β-*tubulin* genotype frequencies in the female worms, recorded after treatment (Figure 1).

### Covariates Associated with Heterozygosity in β-*tubulin* among Female Worms

Before treatment, β-*tubulin* heterozygous status was not influenced by age of host, total number of females in the nodule, total number of palpable nodules or CMFL in the village of residence (Table 3). After treatment, none of the tested covariates was significantly associated with the β-*tubulin* heterozygous status, except the fact of living in a village with a CMFL between 41 and 60 mf/ss. This weak association (OR = 6.24 (95% CI, 1.09–35.59); *p* = 0.039) might indicate that the probability of being heterozygous was higher in the villages where infection rates were rather high (Table 4). The probability of being heterozygous tended to be higher in the groups treated three-monthly. Even if this was not significant in the analysis taking into account the various groups separately, this trend is consistent with the results presented above comparing the pooled annual treatment groups and the pooled three-monthly treatment groups.

**Table 3:** Analysis of the Relationship of β-*tubulin* Genotype with Patient and Parasitological Independent Variables Before Treatment.

*N* = 183 female worms	OR	95% CI	*p*
Age of patient	0.97	0.93–1.00	0.081
No. females/nod.	0.94	0.73–1.21	0.629
CMFL 41–60 mf/ss	0.81	0.13–5.15	0.819
CMFL 61–70 mf/ss	1.93	0.44–8.49	0.385
CMFL 71–114 mf/ss	0.44	0.06–3.15	0.413
No. nod. 1994	1.00	0.81–1.22	0.976

Odds ratios (OR) and 95% confidence intervals (95% CI) for logistic regression of heterozygote status (vs. homozygote) of worms collected before ivermectin treatment on four independent variables. No. females/nod. = total number of females in the nodule; No. nod. 1994 = total number of palpable nodules in 1994; CMFL = Community Microfilarial Load (reference category: 10–40 microfilariae per skin snip (mf/ss)).

**Table 4:** Analysis of the Relationship of β-*tubulin* Genotype with Patient and Parasitological Independent Variables After Treatment.

*N* = 98 female worms	OR	95% CI	*p*
150 µg/kg three-monthly	2.22	0.39–12.65	0.370
800 µg/kg yearly	0.93	0.22–3.94	0.923
800 µg/kg three-monthly	1.68	0.23–12.15	0.609
Age of patient	1.00	0.94–1.04	0.691
No. females/nod	0.83	0.57–1.23	0.356
CMFL 41–60 mf/ss	6.24	1.09–35.59	0.039
CMFL 61–70 mf/ss	4.44	0.91–21.54	0.065
CMFL 71–114 mf/ss*	-	-	-
No. nod. 1994	0.88	0.68–1.13	0.305

Odds ratios (OR) and 95% confidence intervals (95% CI.) for logistic regression of heterozygote status (vs. homozygote) of worms collected after ivermectin treatment on five independent variables. No. females/nod. = total number of females in the nodule; No. nod. 1994 = total number of palpable nodules in 1994; CMFL = Community Microfilarial Load (reference category: 10–40 microfilariae per skin snip (mf/ss)). Reference category for treatment group: 150 µg/kg yearly.

* All worms from villages with CMFL 71–114 mf/ss were heterozygotes; consequently, the highest CMFL category was not used for the estimation of other independent variables.

### Relationship between β-*tubulin* Genotype and Fertility of Female Worms Before and After Treatment

Before treatment, the homozygote genotype was the only independent variable associated with a high fertility phenotype (*p*<0.002) (Table 5). After treatment, high fertility of the worms was still associated with the homozygous genotype (*p* = 0.035). In addition, high fertility of the worms was more likely to be observed amongst younger patients (*p* = 0.018). Finally, high fertility in the worms was more apparent in nodules containing higher numbers of male worms (*p* = 0.030) (Table 6).

**Table 5:** Analysis of the Relationship of Parasite Fertility with β-*tubulin* Genotype, Patient and Parasitological Independent Variables Before Treatment.

*N* = 183 female worms	OR	95% CI	*p*
Genotype = homozygote	3.71	1.59–8.70	0.002
Age of patient	1.00	0.97–1.04	0.940
No. females/nod.	0.85	0.70–1.02	0.085
CMFL 41–60 mf/ss	0.69	0.15–3.23	0.635
CMFL 61–70 mf/ss	1.12	0.25–5.09	0.879
CMFL 71–114 mf/ss	1.93	0.40–9.37	0.415
No. nod. 1994	0.97	0.86–1.10	0.633
No. males	1.10	0.76–1.60	0.599

Odds ratios (OR) and 95% confidence intervals (95% CI) for logistic regression of full fertility (vs. low- or non-fertility) of worms collected before ivermectin treatment on six independent variables. Reference category for genotype: heterozygosity for β-*tubulin* gene. No. females/nod. = total number of females in the nodule. No. nod. 1994 = total number of palpable nodules in 1994. No. males = number of males in the nodule. CMFL = Community Microfilarial Load (reference category: 10–40 microfilariae per skin snip (mf/ss)).

**Table 6:** Analysis of the Relationship of Parasite Fertility with β-*tubulin* Genotype, Patient and Parasitological Independent Variables After Treatment.

*N* = 98 female worms	OR	95% CI	*p*
Genotype = homozygote	3.90	1.10–13.79	0.035
150 µg/kg three-monthly	1.71	0.27–10.60	0.566
800 µg/kg yearly	0.78	0.20–3.14	0.731
800 µg/kg three-monthly	2.47	0.42–14.58	0.320
Age of patient	0.94	0.89–0.99	0.018
No. females/nod.	1.10	0.76–1.76	0.506
CMFL 41–60 mf/ss	0.33	0.04–3.21	0.342
CMFL 61–70 mf/ss	1.59	0.20–12.47	0.657
CMFL 71–114 mf/ss	0.57	0.06–5.18	0.619
No. nod. 1994	1.09	0.84–1.40	0.522
No. males	2.38	1.09–5.21	0.030

Odds ratios (OR) and 95% confidence intervals (95% CI) for logistic regression of full fertility (vs. low- or non-fertility) of worms collected after ivermectin treatment on seven independent variables. Reference category for genotype: heterozygosity for β-*tubulin* gene. Reference category for treatment group: 150 µg/kg yearly. No. females/nod. = total number of females in the nodule; No. nod. 1994 = total number of palpable nodules in 1994; No. males = number of males in the nodule; CMFL = Community Microfilarial Load (reference category: 10-40 microfilariae per skin snip (mf/ss)).

**Figure 2:**
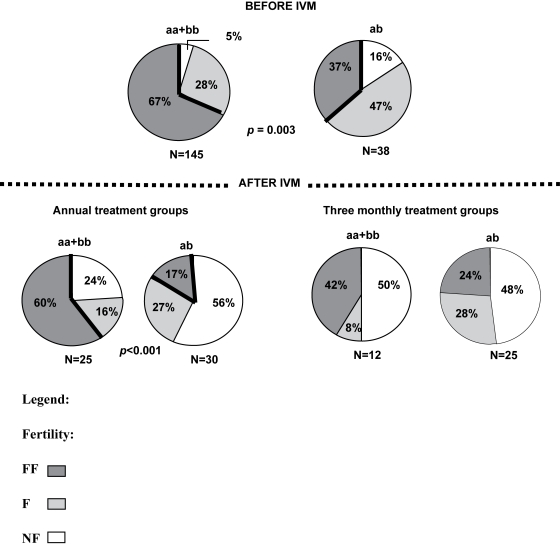
Relationship between Female Worm Fertility and β-*tubulin* Genotype in Relation to Treatment Group. Relationship between female worm fertility and β-tubulin genotype (homozygous or heterozygous) in relation to treatment group (annual versus three-monthly treatments, corresponding also to 4 versus 13 treatment rounds within three years). IVM = ivermectin treatment; FF = fully fertile; F = low fertility; NF = not fertile.

As the intervals between the last IVM treatment and nodulectomy in the three-monthly groups and the annual treatment groups were different, they have also been considered separately (Figure 2). Twelve months after the last IVM treatment, analysis of the fertility (non- and low fertility versus full fertility) in relation to genotype showed that the β-*tubulin* homozygous worms remained more fertile than the heterozygous worms (χ^2^ = 11.06, *p*<0.001; Figure 2). Because the sample size was small, we did not perform an analysis on the data collected on the three-monthly groups (samples collected three months after the last IVM treatment; groups 2 and 4). However, the figure shows that the proportion of fully fertile worms was higher in the homozygous worms (42% compared with 24%), but both genotype groups showed a similar proportion of non-fertile worms (50% and 48%, respectively, for the homozygous and heterozygous parasites).

## Discussion

With the implementation of the onchocerciasis control programmes, an increasing proportion of people in endemic areas have received community-directed treatment with IVM on a regular basis. Even though children under 5 years of age and pregnant women are excluded from mass treatment, a high proportion of the parasite population, in control areas, is under treatment. As a consequence, and because most of the parasite population is in the human hosts rather than in the vector, only a relatively small proportion of the *O. volvulus* population is likely to be *in refugia* (not exposed to the drug) at the time of treatment. Thus, selection pressure for any IVM resistance alleles is expected to be high in *O. volvulus* [7].

Parasitological and epidemiological evidence of ivermectin resistance in *O. volvulus* populations has been reported in Ghana [11]. Selection on the β-*tubulin* gene following repeated IVM treatment of people infected with *O. volvulus*, compared with parasites from IVM-naïve people has been found in Ghana [16,17]. Because gene selection is the first step in the development of drug resistance, it is important to assess genetic change in a population of parasites exposed to selection pressure.

Our study of the possible effects of IVM on the genetic structure of an *O. volvulus* population is unique in several respects. We analyzed nodules from the same patients before and after three years of IVM treatment at different treatment frequencies, dose rates, and with complete knowledge of the number of IVM treatments. To our knowledge, this has never been done in past investigations of the effect of IVM on genetic selection in human parasitic nematodes. In the study area, because there was no vector control and only a small proportion of the population living in the area was treated during the trial, the force of infection probably did not decrease during the trial [3].

The main finding, that IVM treatment selected for heterozygotes at the β-tubulin locus and that this selection was dependent on the number of doses, raises interesting questions in view of the fact that this gene has been linked with IVM resistance in another parasitic nematode [17] and the recent evidence that IVM resistance is occurring in *O. volvulus* [11]. The period over which the IVM treatment-associated genetic change in β-*tubulin* occurred was short (1994 to 1997). It takes about 1 year from microfilarial birth until an adult worm commences production of the next generation of microfilaria. The observed genetic changes are dramatic, given the time period and the generation interval, and could result from differential mortality of existing adult worms and possibly a differential establishment of new worms, dependent on the worm genotype and tolerance to the drug pressure. These possible selective events will require further investigation. IVM might be more toxic to the more fertile female worms, which were previously found to be homozygous at the β-*tubulin* locus [18], as a result of the effect of IVM in preventing the release of microfilariae from the uterus and the subsequent degeneration of these trapped microfilariae.

The main limitation of the present study was that some samples could not be genotyped. As suggested in the previous paper [18] with samples from the same trial [3], there are several explanations for the difficulty in genotyping some of the adult worms. The nodules had been stored in a dessicating fixative, at room temperature, for several years before they were digested and the DNA extracted. It is likely that some of the worms that could not be genotyped were dead or moribund at the time that the nodules were harvested. The DNA of dead or moribund parasites may have been degraded or fragmented, and difficult to amplify. Dead *O. volvulus* are not rapidly resorbed and can be readily found in nodules [20,23]. In the study area before treatment, 15.2% of adult female worms found in nodules were moribund or dead [24]. After treatment, the proportion of non-genotyped parasites was higher (*p* = 0.024), which could be due to the macrofilaricidal effect of IVM [3,^25^,26].

Very similar numbers of worms were genotyped for β-*tubulin* and for *hsp60* before and after treatment. Most of the worms that could not be genotyped for the acidic ribosomal protein gene could also not be genotyped for β-*tubulin*. However, a higher proportion of the *acidic ribosomal protein* gene was genotyped compared with the β-*tubulin* and the *hsp60* genes. The fact that *acidic ribosomal protein* has many gene copies in eukaryotic genomes [21], whereas β-*tubulin* and *hsp60* typically do not occur with multiple gene copies, could explain the difference in the ease of genotyping the *acidic ribosomal protein* gene compared with the β-*tubulin* and *hsp60* genes.

Notwithstanding these likely reasons why some worms could not be genotyped, we have performed several analyses to evaluate whether the population of those worms that could not be genotyped was similar to the population of worms that were genotyped. We showed that the two populations did not differ according to various external covariates (host age, CMFL level, etc.).

The female worms were not dissected into different tissues, but each worm was analyzed as a single entity. Any microfilarial DNA present within the female worm would be included in the DNA analysis of the female worm, and so the β-*tubulin* genotype frequencies of the female worms could have resulted from microfilarial/embryo DNA contamination within the female worm. Such daughter microfilariae/embryos would have half of their DNA derived from that mother worm and thus reflect the genome of the mother worm. Any contribution from male worms to the assessed genotype of a female worm via the daughter microfilaria/embryos contained within the female worm, would, if a significant effect, tend to increase the probability that female worm genotypes would be seen as heterozygotes. In fact, in the female worms before treatment there was an excess of homozygotes, indicating that any contribution from male worms to the genotype assessed in the female worms was not significant. Given the low frequency of the “b” allele in the male worms before and after treatment, the increase in the frequency of “ab” heterozygotes in the female worms, following treatment, could not be accounted for by male worm contamination via microfilaria/embryos contained within the female worm.

Finally, the frequency of “ab” heterozygotes was greater in the three-monthly treatment groups (69%) than in the annual treatment groups (56%) and the pre-treatment sample (21%). IVM treatment reduces fertility so that if microfilarial/embryonic DNA were contributing to the assessment of DNA of the female worms, treatment should have decreased, rather than increased, the proportion of female worms that appeared as heterozygotes. For these reasons we do not believe that microfilarial/embryonic DNA within the female worms had any significant influence on the observed genotype frequencies for β-*tubulin* observed in the female worms.

One possible explanation for the apparent IVM selection on β-*tubulin* in the female worms is the existence of a null allele (an allele that is not being detected by the method used) that could be distorting the genotype frequencies determined. Previously, we have found that freshly frozen *O. volvulus* samples always amplified readily with the same β-*tubulin* primers as have been used in this study [17] suggesting that no null allele exists, using the procedures followed, for β-*tubulin* in *O. volvulus*. We do not believe that a null allele exists to affect the genotype frequencies. Furthermore, even if it did exist, it could not account for the change in genotype frequencies resulting from IVM treatment. We can conclude that the observed change in β-*tubulin* genotype frequency with treatment is genetic selection as has been seen previously in *O. volvulus* that have been obtained from people who have been repeatedly treated with IVM and also observed in IVM-resistant *H. contortus* [16,17].

One of the main findings of the study is that female worms, homozygous for β-*tubulin*, were more fertile than heterozygote female worms. A similar difference in fertility before treatment was reported previously [18]. Homozygous worms, in the patients treated annually and collected twelve months after the last IVM treatment, also had higher fertility than heterozygotes. However, this difference decreased if the worms were collected three months after 13 three-monthly IVM treatments, at a time when the embryostatic effect of IVM is normally still apparent. The results observed three months after IVM (Figure 2) could be due to a combination of innate differences in fertility between homozygous and heterozygous worms and the relative effects of IVM on embryostasis in the different genotypes. If the fertility disadvantage of heterozygotes tends to disappear when the parasite is under strong IVM pressure, this could have implications for parasite transmission and possible resistance selection. It would be interesting to study, perhaps in *Caenorhabditis elegans*, how polymorphism in β-*tubulin* may affect the fertility of nematodes.

Before treatment, *hsp60* gene was in Hardy-Weinberg equilibrium in the female and male worm populations as well as for the β-*tubulin* gene in the male worm population. The *acidic ribosomal protein* gene normally has multiple gene copies [21], which means that there are multiple loci in the genome. For this reason, it is inappropriate to apply a Hardy-Weinberg equilibrium test, as used on a single locus. The β-*tubulin* gene was not in Hardy-Weinberg equilibrium in the female worms, as there was an excess of homozygotes in this population. Various assumptions are required for Hardy-Weinberg equilibrium including random mating. Non-random mating could occur in the *O. volvulus* population and be due to inbreeding, positive assortative mating or a subpopulation structure [27]. The inbreeding coefficient *F*, which represents the proportional loss of heterozygosity due to inbreeding, was calculated for the population of 73 patients, based on the β-tubulin data, to be 0.41. This index of inbreeding is moderately high and has implications not only for Hardy-Weinberg equilibrium, but also for the possible rate of selection of a resistant population [28]. Inbreeding could be explained by the fact that vectors transmitting to the study population were living in the local area and tend to bite people from the same community.

It is of interest that the *F_IT_* (inbreeding coefficient within a subpopulation) for *Wuchereria bancrofti* microfilariae, a closely related human filarial nematode, was calculated on the basis of β-*tubulin* genotype frequencies to be 0.44 [29]. However, other processes may be involved. An assortative mating coefficient R (which could include inbreeding) was calculated [27] to be 0.54. The higher fertility of the female β-*tubulin* homozygote “aa” worms, when compared with the female heterozygote “ab” worms, could be consistent with positive assortative mating if the likelihood that a worm will mate is associated with the female worm's fecundity. It is known that male *O. volvulus* migrate between nodules and may be attracted by the fully fertile females (predominantly homozygous). Subpopulation structure could also explain the Hardy-Weinberg disequilibrium. Because the samples were collected in a forest/savannah transition zone, the worms could belong either to a savannah, or to forest or mixed forest/savannah strains. Subpopulations can result from environmental segregation, inbreeding and/or positive assortative mating. Finally, it is possible that in the absence of IVM treatment, the β-*tubulin* heterozygote female worms die faster than the homozygote female worms. Differential mortality between the genotypes could also affect Hardy-Weinberg equilibrium.

The selection for the β-*tubulin* heterozygote “ab”, found in female worms after IVM treatment, was more important in the worms exposed to IVM every three months compared with the worms exposed to IVM annually. This difference could be due to either the total number of treatments or to the interval between treatments. These are important points for the onchocerciasis control programmes because semi-annual or more frequent treatments are ongoing in some areas and under consideration in other areas. An increase in treatment frequency might increase the selection pressure.

No selection was demonstrated in the male worm population after treatment, during the three-year study period. This result suggests that the female worms are more susceptible to IVM pressure than the males and could reflect an increased IVM-induced mortality in female worms that are homozygous at the β-*tubulin* locus. From the Gardon et al. trial in Cameroon [3], it is possible to estimate the relative proportional decrease in male and female parasites due to IVM treatment over the study period. This amounts to 13.5% and 27.5%, respectively. If the male parasites are less affected by the treatment, the genotype frequencies in the male population would only change significantly following transmission and subsequent infection by progeny of the parasites under selection. Given that it takes about one year to complete the life cycle of *O. volvulus*, in this situation it would take longer for IVM selection to influence male worm genotype frequencies. It is interesting in this regard that in *Brugia malayi*, sex-dependent expression in a possible IVM receptor has been demonstrated [30]. The putative glutamate-gated chloride (GluCl) channel gene, *AF118554,* was estimated to be expressed at a 24.3-fold higher level in female worms compared with male worms. As GluCl is thought to be the main target of IVM [31], one could speculate that the effect of IVM might be greater in female than male worms. This might explain why *O. volvulus* male worms seem to be less susceptible to IVM and thus less rapidly selected than female worms, so that in the short three-year time interval of this study no significant change in β-*tubulin* genome frequency was seen in the male worms. However, selection on β-*tubulin* in male *O. volvulus* has been found when parasite populations were exposed over 6 or more years to IVM [16,17].

There may also be an IVM-induced mortality of the incoming larvae or pre-adult stages of the parasites, with the homozygous worms being more sensitive to repeated treatment than the heterozygote parasites. An effect of IVM on the pre-adult stages of *O. volvulus* has been suggested [32] and demonstrated in results obtained on the bovine parasite *Onchocerca ochengi* [33].

Today, intestinal trichostrongylid nematodes of livestock are commonly strongly resistant to IVM, and the development of IVM resistance can occur rapidly in these nematode parasites, sometimes in less than three years [34]. However, trichostrongylid nematodes and filarial nematodes have different biology. As the generation time is shorter in trichostrongylid nematodes than in filarial nematodes, resistance selection would be expected to take longer to be manifested in filarial worms. In contrast to soil transmitted nematodes, filariae have no free-living stages and most of the population of the nematode occurs in the human host, so that *refugia* is likely to be low in a community under treatment. As a result, selection pressure for resistance to develop could be high in human filarial nematodes under intensive drug treatment [7].

### Conclusion

IVM has been used since the late 1980s, and more than 400 million doses have been distributed in Africa [4]. It remains the only safe drug for community treatment of onchocerciasis. Our results clearly show a genetic selection in *O. volvulus* caused by repeated IVM treatment. Since the parasites were collected before and after treatment from the same patients, these results cannot be explained as differences arising from different host populations being sampled. These results, together with other evidence of genetic selection and reports of sub-optimal responses to IVM, provide a warning that selection for IVM resistance could be occurring in some populations of *O. volvulus*.

In view of these results, it is imperative that field studies be undertaken to characterize all treatment responses to IVM in *O. volvulus*, coupled to further genetic analysis, in order to confirm or not the possible emergence of IVM resistance. Such longitudinal studies, which would look at the repopulation of the skin of treated people by mf, should be undertaken without delay if the benefits that have been achieved by the onchocerciasis control programmes are not to be lost as a result of the spread of IVM resistance in *O. volvulus*.
